# Application of botulinum toxin in maxillofacial field: part I. Bruxism and square jaw

**DOI:** 10.1186/s40902-019-0218-0

**Published:** 2019-10-01

**Authors:** Kyung-Hwan Kwon, Kyung Su Shin, Sung Hee Yeon, Dae Gun Kwon

**Affiliations:** Department of Oral and Maxillofacial Surgery, College of Dentistry, Wonkwang University, Iksan, South Korea

**Keywords:** Botulinum toxin, Clinical application, Maxillofacial field, Bruxism, Square jaw

## Abstract

The application of botulinum in oral and maxillofacial surgery begins in 1982, where Jan Carruthers started using it for reducing the muscle mass and smoothing the skin, and since then it has been used for cosmetic purposes. In Korea, it is already being used by various specialties including dentistry (oral and maxillofacial surgery, oral medicine), plastic surgery, dermatology, ophthalmology, general surgery, and orthopedic surgery, etc. Each specialty approaches to Botox with its own medical indications. In this article, we will discuss the maxillofacial application of botulinum toxin, which includes theoretical and practical aspects of such as bruxism and square jaw.

## Background

Nowadays, an agent used to smooth the facial wrinkles and fine lines, Botox (botulinum toxin A), is of great attention in the dentistry field. Botox is the commercial name for botulinum toxin. It is like we simply call acetaminophen as Tylenol. Botulinum toxin is known to be four times more toxic than common tetanus toxin, and more than ten times toxic than curare. Since its introduction in the plastic surgery for cosmetic use in the 1980s, it is being widely used in various fields including dentistry, dermatology, ophthalmology, plastic surgery, general medicine, etc. [1]. At present, botox under the brand names BTXA (Hanall pharmaceuticals, China), Dysport (Beaufour Ipsen Korea (Ltd), France), and Botox (Daewoong Pharma Importer, Allergan, USA) are marketed in Korea. Commercially available forms that we commonly use are of serotype A; serotype B was released in the USA under brand name Myoblock but it is not available in Korea [2].

The therapeutic effect of botulinum toxin is due to its action on neuromuscular junction. It induces flaccid paralysis by inhibiting acetylcholine release [3]. Mechanism of action consists of three stages: binding, internalization (energy-dependent receptor-mediated endocytosis), and flaccid paralysis through inhibition of releasing neurotransmitter [2]. This therapeutic effect continues for 3–6 months; within that period, botulinum toxin corrects the patterns of muscle exercises, decreases facial wrinkles or square jaw, and alleviates pain by changing the patient’s lifestyle [4].

## Background

### History of botulinum toxin

Justinus Kerner had discovered toxin from rotten sausages and reported in 1829. In 1897, Professor Emile Pierre van Ermengen from Belgium discovered anaerobic bacteria capable of forming spores from salted pork meat and from a cadaver infected with botulinum toxin (botulism) [5]. Since then, this bacteria is named as *Clostridium botulinum*, and exotoxin protein BTX-A, which this bacteria expresses was named after the bacteria [6]. With the outbreak of World War I and World War II, people refined botulinum for weaponization, and scientists started to research using refined BTX-A to study its mechanism of action, and its action on the contracting mechanism of muscle. In 1973, Alan B. Scott was the first to use BTX-A for treating strabismus. Since 1979, as the FDA (American Food and Drug Administration) approved BTX-A for treating strabismus; it is being widely used for various treatment purposes [1]. After then, clinical studies were performed on blepharospasm or hemifacial spasm, and in 1989, the FDA finally approved its application in these diseases as well.

The application of botulinum in oral and maxillofacial surgery begins in 1982, where Jan Carruthers started using it for reducing the muscle mass and smoothing the skin, and since then it has been used for cosmetic purposes. It was in 1990 that the use of Botox in head and neck area began, first used for a bruxism patient with brain injury [7]. Smyth had observed the remarkable effects in patients with square jaws (bilateral masseteric hypertrophy) [8]. According to Freund in the 2002 journal, he observed good effects when he used Botox in TMJ patients [4]. On December 8, 2001, Dr. Kerusus gave a lecture on wrinkle removal procedure of Botox in Korea, and in 2002, the FDA approved the use of Botox for cosmetic purpose. In Korea, it is already being used by various specialties including dentistry (oral and maxillofacial surgery, oral medicine), plastic surgery, dermatology, ophthalmology, general surgery, and orthopedic surgery, etc. Each specialty approaches to Botox with its own medical indications.

### Comparison of commercial products of type A botulinum toxin

Botox has 100 allergan units of toxin A per vial, and Dysport has 500 Speywood units of toxin A per vial. In this article, the units of Botox and BTX A will be expressed as botox unit (BU) and the unit of Dysport as DU. Equivalent effect of the toxin is known to be about 1 BU = 3–4 DU. Dose for cosmetic purposes is 0.5–1 BU/kg, and dose for spasticity is 15–18 BU/lg. LD50 (lethal dose) of 70 kg person is found to be 2800 BU, therefore the dose seems to be relatively safe. In other words, you need as many as 28 bottles administered at once for it to be lethal since one bottle of Botox contains 100 BU (Table 1).

**Table 1:** Comparison of Botox and Dysport

	Botox	Dysport
Components	Clostridium botulinum toxin	Clostridium botulinum toxin
Vial	Type A 100 U	Type A 500 U
Effects	1 Botox unit	3~4 Dysport unit
Half-life	Dry: 24 months Solution: 5 h	Dry: 1 year Solution: 8 h
Manufacturer	Allergan, Irvine, CA, USA	Ipsen, Ltd, Wrexham, UK
Dilution	100 Unit + 2.5 ml NaCl Physiologic solution : 2 U in 0.05 ml : 4 U in 0.1 ml	500 Unit + 2.5 ml NaCl Physiologic solution : 10 U in 0.05 ml : 20 U in 0.1 ml

### Method of diluting botulinum toxin

Method of diluting botulinum toxin varies depending on the surgeon. Generally, in the treatment of square jaw, we use BTXA, Botox, and Dysport. For Botox, We dilute it with 2.0 cc saline or distilled water. It is 5 BU per 0.1 cc, so it is easy to measure (for 2.5 cc normal saline, you can calculate as 4 BU per 0.1 cc). For Dysport, We dilute it with 2.5 cc saline or distilled water, it is 20 DU per 0.1 cc, and therefore you can easily adjust the amount to be injected into the masseteric muscle [1, 7–10].

**Fig. 1:**
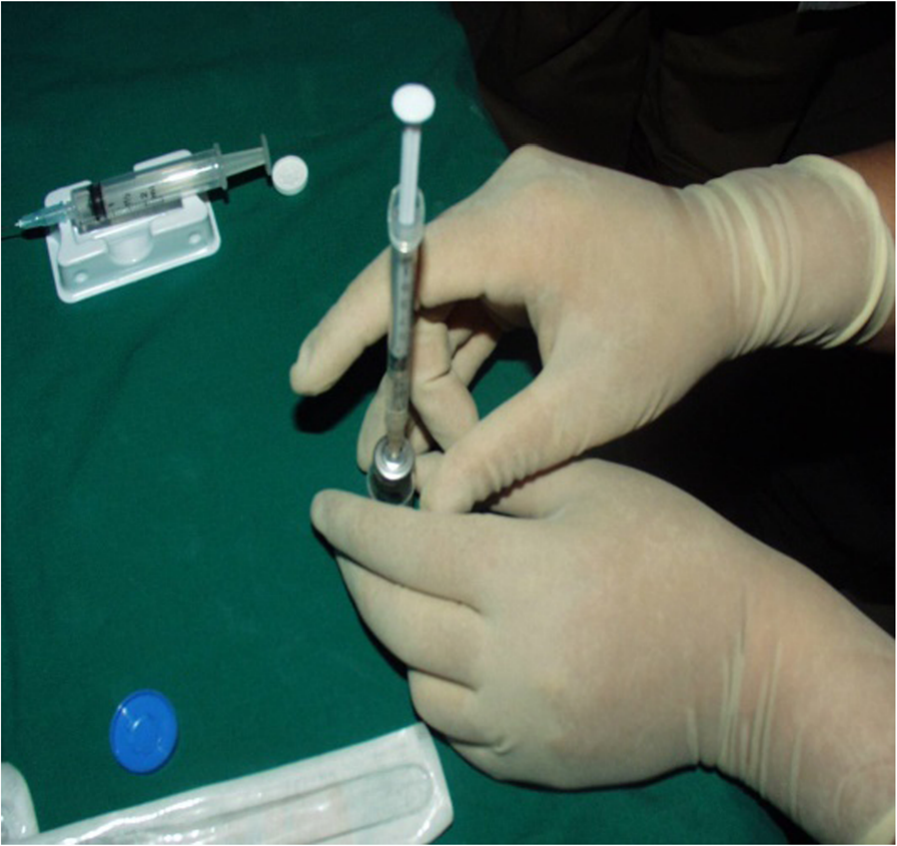
Injecting 2.5 cc saline into botulinum toxin vial

**Fig. 2:**
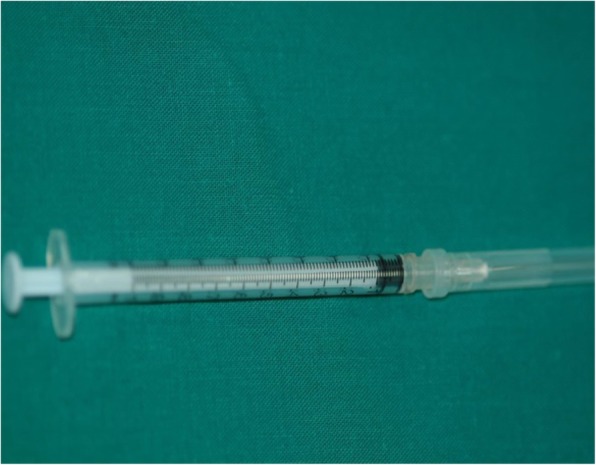
1 cc insulin syringe

Using a 5 cc syringe, draw more than 3 cc of air and inject into the botulinum toxin vial. Check whether the air is sucked into the vial. If air is not sucked into the vial, discard the vial as the vacuum is not present in the vial. To achieve a desirable concentration of dilution solution, prepare for each normal saline. We normally prepare for 2.5 cc. Inject 2.5 cc of normal saline into botulinum toxin and then ‘gently’ mix them (on shaking, disulfide bond consisting of heavy chain and light chain breaks off and reduces the therapeutic effect). In this way, you can dilute the botulinum toxin and it is ready for use for injection with a 1 cc syringe (Figs. 1 and 2).

### Scope of application of botulinum in Maxillofacial field

Glabellar lines, wrinkles resembling Crow’s feet around the eyes, wrinkles formed around the eyes while smiling, deep parallel lines of the forehead, and square jaw are the most common indications for botulinum toxin injection. The scope of advanced application include bruxism, enhancement of wound and skin, softening of skin and correction of scars, treatment of keloid, treatment of tension headache in patient with temporomandibular joint disorder, and it is also used to reduce maximum bite force in early loading of an immediate implant. In Korea, the study on its application in trigeminal neuralgia and stomatitis pain reduction is in progress [9, 11].

### Determination of dose depending on the treatment area

**Fig. 3:**
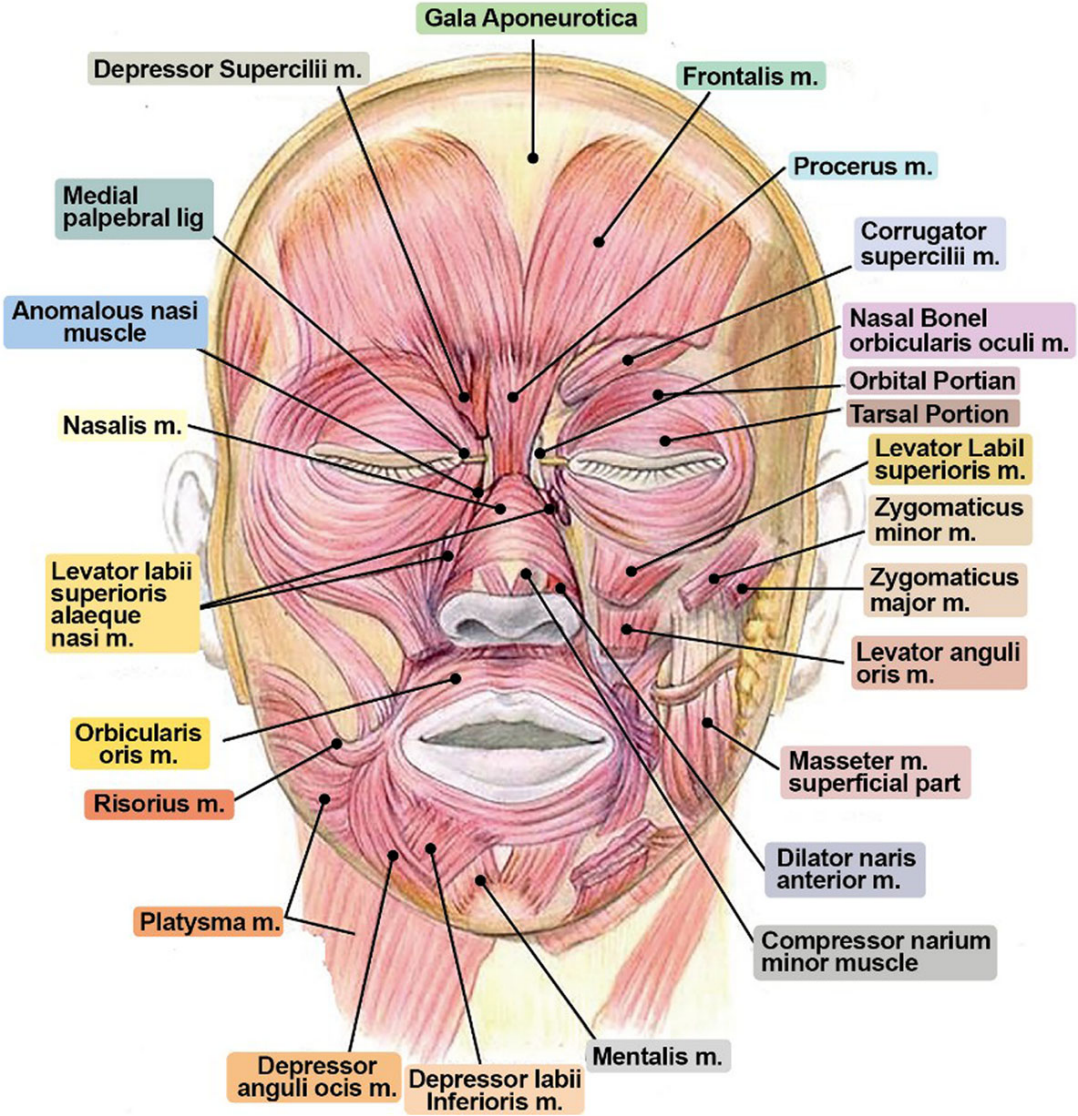
Perform botulinum toxin injection after understanding the overall anatomical structures for facial muscles. In particular, carefully look for orientation of frontalis, procerus, and masseteric muscle

**Fig. 4:**
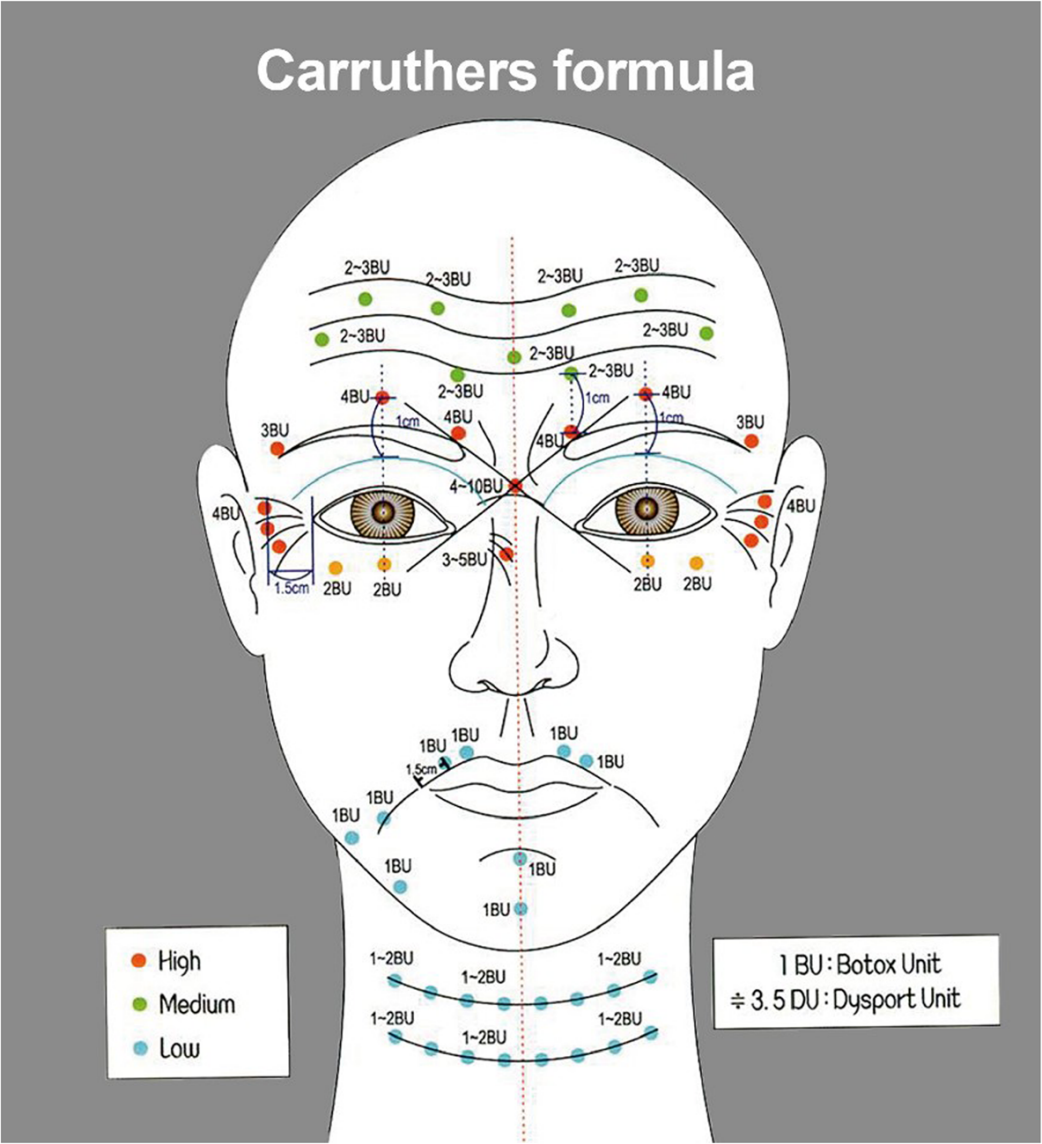
A protocol used for forehead wrinkles, eye wrinkles, etc. A standard dose is estimated based on the dose of Botox and BTXA (formula by Carruthers) [12, 13]

Total dose injected into each area may vary depending on the operator. In this article, we will recommend the dose for each treatment area based on our clinical experience. For glabellar lines and wrinkles around the eyes, inject 2–4 BU (8–10 DU) per one injection point. For forehead, inject 4–5 points, inject 2 points for glabellar lines and 3 points for wrinkles around the eyes. Eventually, the total amount required for the treatment will be 10–35 BU for glabellar lines, 12–20 BU for the forehead, and 12–24 BU for eye wrinkles. For square jaw, inject 8–10 BU per point into masseteric muscle. Inject 3 to 4 points. Toxin spreads easily by penetrating the tissues, and fascia reduces the diffusion of the toxin. On injecting 10 BU of Botox or BTXA, relaxation occurs within the circular region with 3 cm in diameter from the injection point (Figs. 3 and 4).

**Fig. 5:**
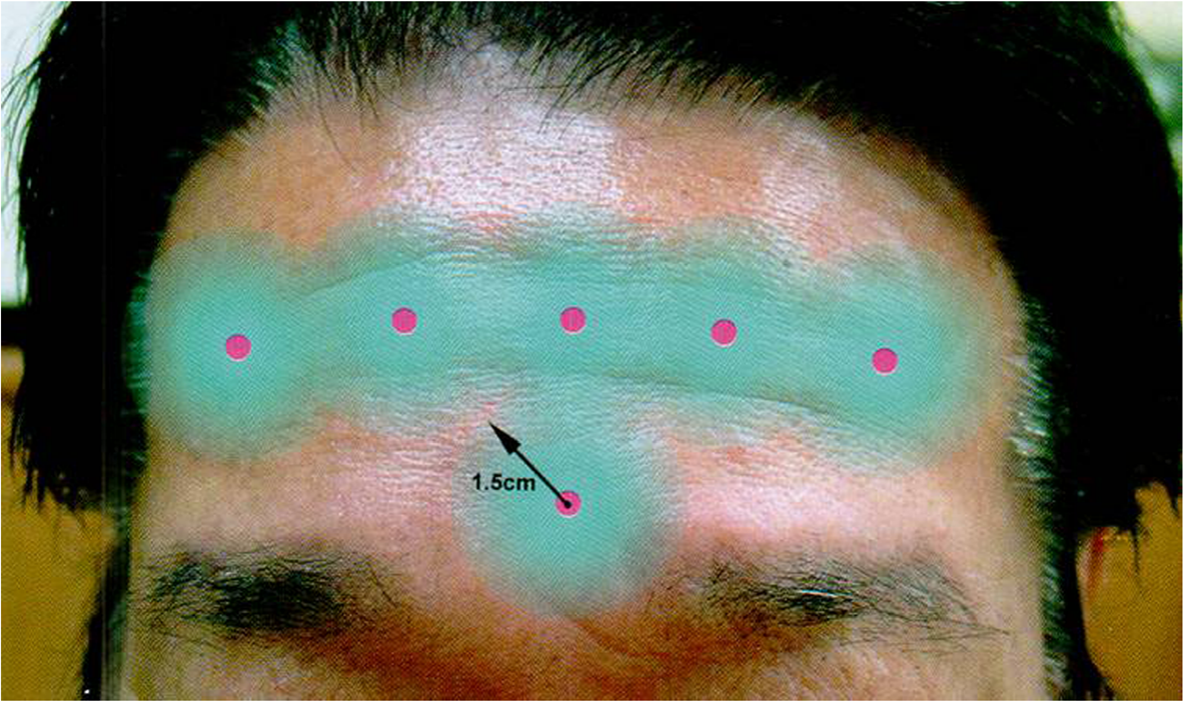
When injecting botulinum toxin in the forehead wrinkles, noted that the injecting point should be 1.5–2 cm far away from the eyebrows

Since toxins evenly spread 2.5–3 cm from injection points, injections should be done at a distance from the area where important nerves arise from. In the case of forehead wrinkles, the injection should be done at least 1.5 cm away from the eyebrows to avoid drooping or swelling of the eyelid (Fig. 5).

**Fig. 6.**
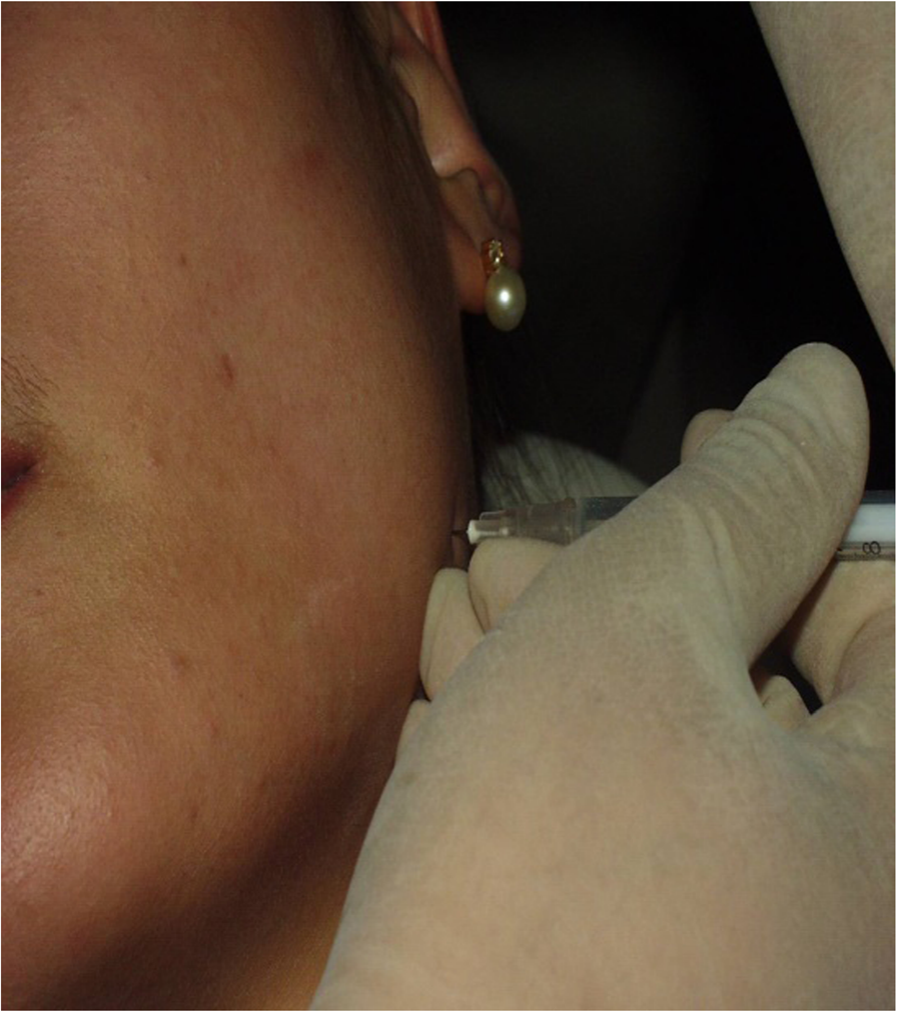
In the case of a square jaw patient, inject the toxin 1.5–2 cm far away from the margin of the mandible. Inject the toxin into the center of the triangle formed with a line joining corner of the mouth and tragus of the ear with a line joining the angle of the mandible and corner of the mouth

**Fig. 7:**
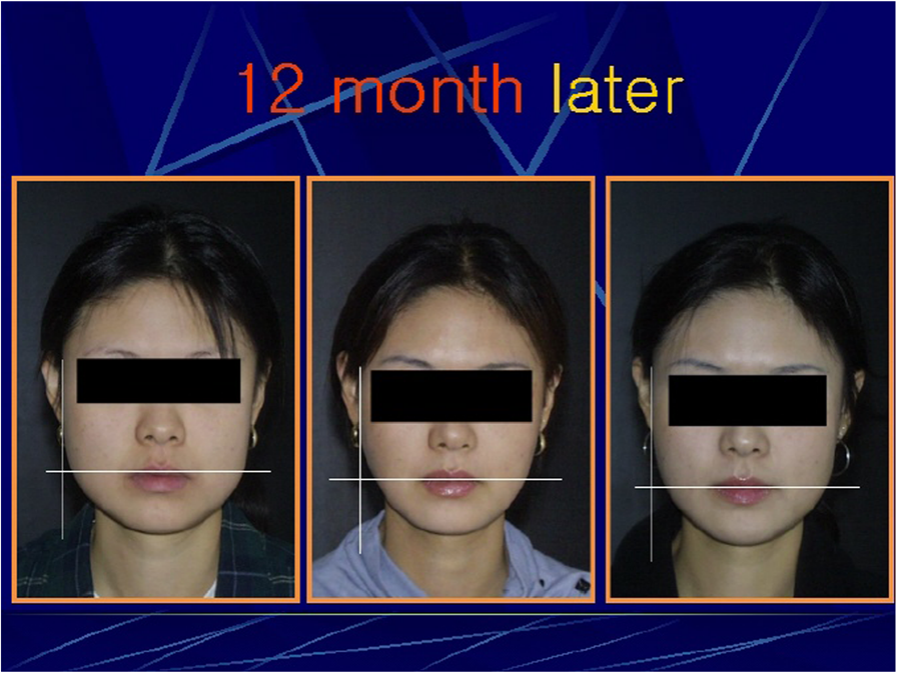
Picture in the left is before the injection, picture in the middle is after 6 months of injection, and picture in the right is after 12 months of injection. The therapeutic effect was greatest at 6th month, and even though there was a bit of regression phenomenon at 12 month compared with the initial picture one can able to continuously observe contraction of the masseteric muscle. It is possible to determine the timing of the second injection.

In the case of square jaw treatment, the injection should be done at a distance of 1.5 cm or more from the lower border of the mandible, and injecting into facial nerves should be avoided (Figs. 6 and 7). The dose injected into one masseteric muscle is 30 BU, for Dysport, it is 100 DU per one side, and the total dose recommended for one person is 200 DU. The number of injections to each mandible is three to four times, and 10 BU per injection (which is equivalent to 0.2 cc when diluted in 2.0 cc saline). Care should be taken while using Dysport, as it is 20 U per 0.1 cc when diluted in 2.5 cc saline.

Since complications often occur due to diffusion of toxin, dose, the direction of the injection needle, and bleeding on injection, you should take these into consideration while injecting. By doing so, you can minimize the occurrence of blepharoptosis, drooping lips, and paralysis of facial muscles resulting from diffusion at major nerve serving areas. Possible local complications include pain, edema, erythema, ecchymosis at the site of injection, headache, short-term hyperesthesia, paresthesia, etc. [5]. Not too much worry about it, as you can prevent all these complications by correctly injecting the needle, slowly injecting the solution, leaving ice-packs on the affected area, and gently massaging the area (avoid rubbing). Avoid taking aspirin or aspirin-containing non-steroidal anti-inflammatory drugs (NSAIDs) 4–7 days before injection to minimize bruising. Moreover, aminoglycoside class of antibiotics or drugs of neuromuscular blockade may enhance the effect of botulinum toxin [10].

**Fig. 8:**
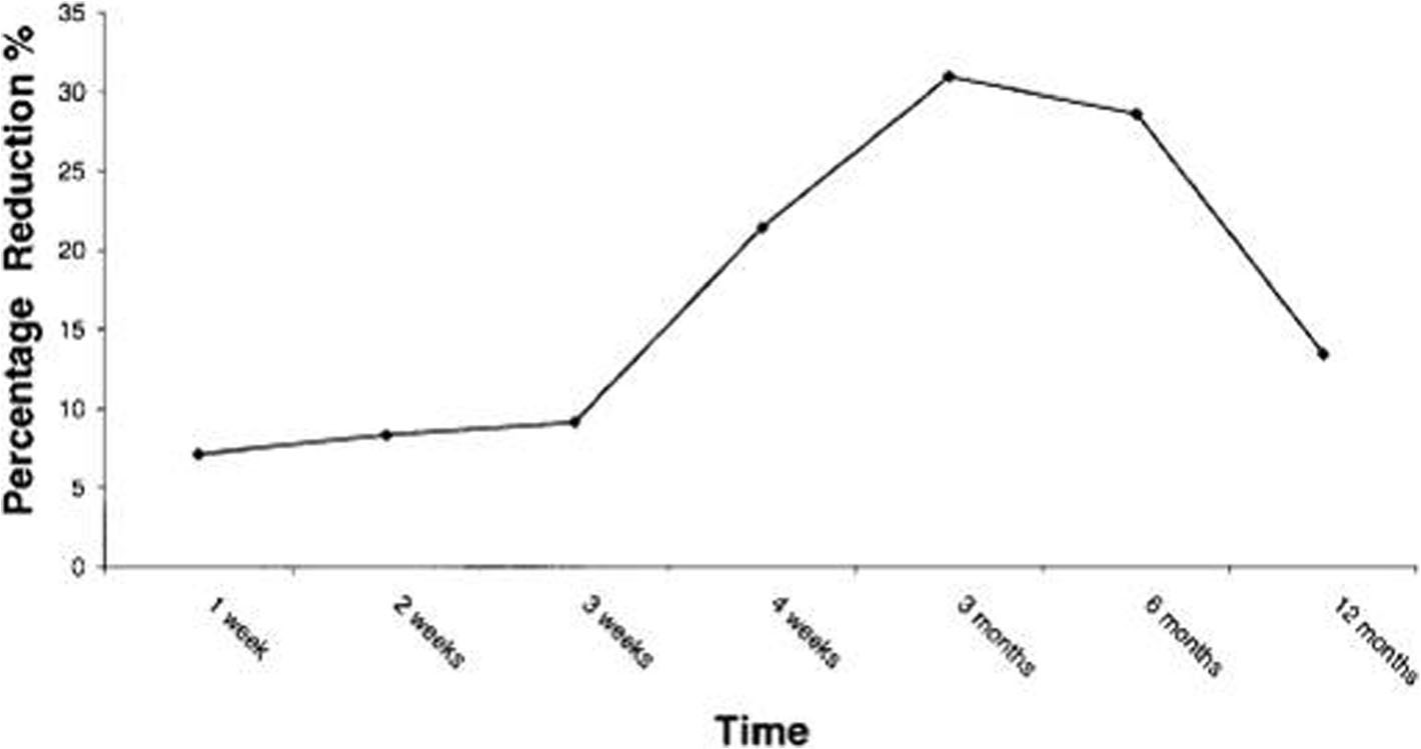
Variation in percentage reduction in muscle mass over time. This graph demonstrates the variation in percentage reduction in muscle mass overtime. According to the result of ultrasonic testing, the extent of reduction in muscle mass began after the injection, from third week, and persisted up to 6 months. It is evident that the second injection should be performed within 3–6 months [14]

**Fig. 9:**
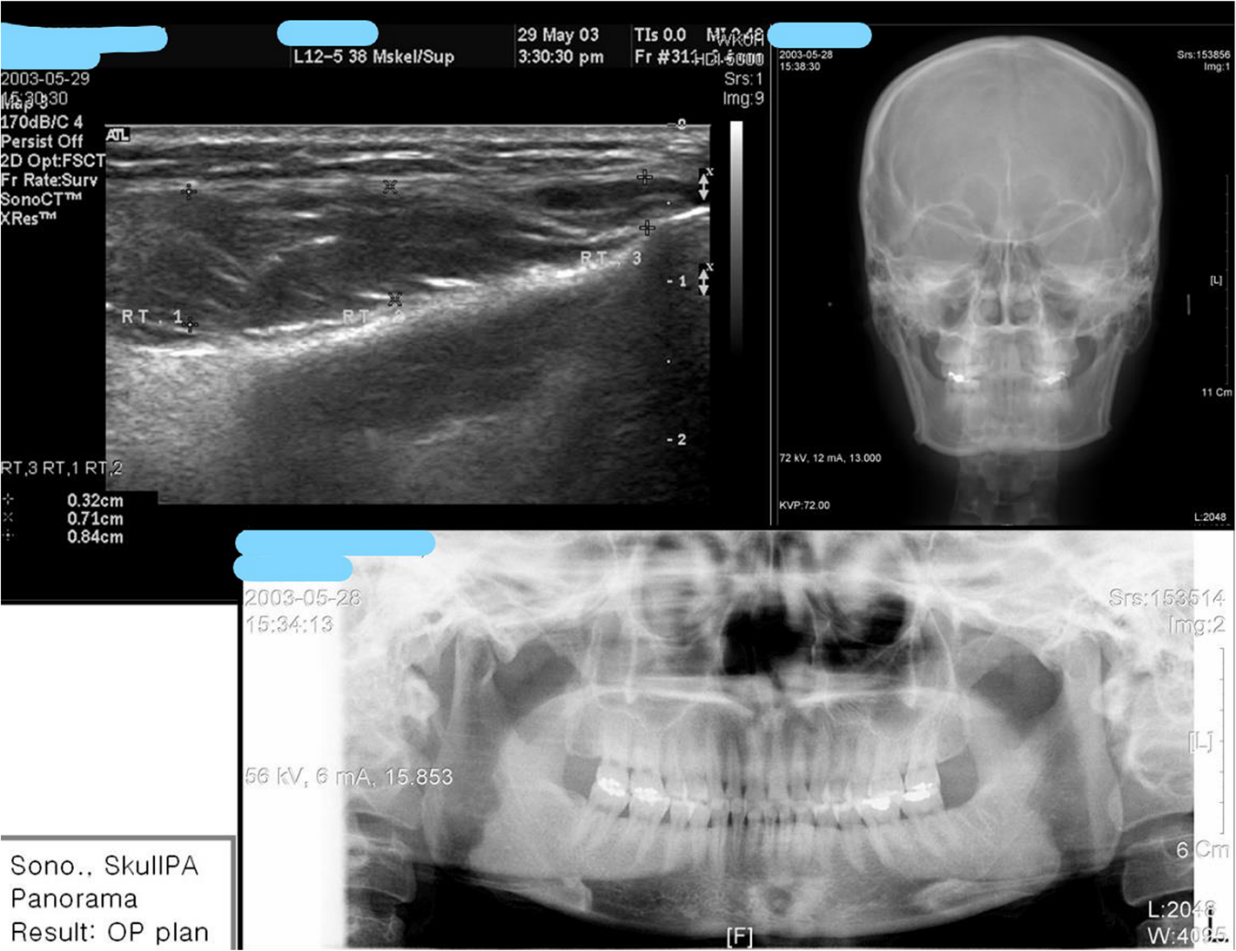
For an initial assessment of bruxism and square jaw, with tests such as sonography, skull PA, and panorama as seen above, one can examine the degree of contraction or hypertrophy of masseteric muscle of a patient with bruxism or square jaw, and able to identify the temporomandibular joint disorder, determine occlusal relationships, and diagnose pathological diseases. An average of 10 mm or more is determined as masseteric hypertrophy

When the efficacy of botulinum toxin was evaluated according to the masticatory force and the degree of contraction of the masticatory muscle, the average reduction of 20% during the 6 months was maintained and the contraction of the masticatory muscle was maintained at about 30% for 6 months (Fig. 8) [14]. We are going to prepare a standard for judging and evaluating the contraction of the masticatory muscle through ultrasound photographs easily accessible in clinic rooms or in dental clinics. We are currently doing research on the use of ultrasound photographs to determine the degree of masseteric muscle hypertrophy and the thickness of the masseteric muscle of the general adult men and women (Fig. 9). After this research, we expect that we would be able to set a standard for the square jaw, find out the dose, method of evaluation, and the timing of the second injection [15].

**Fig. 10:**
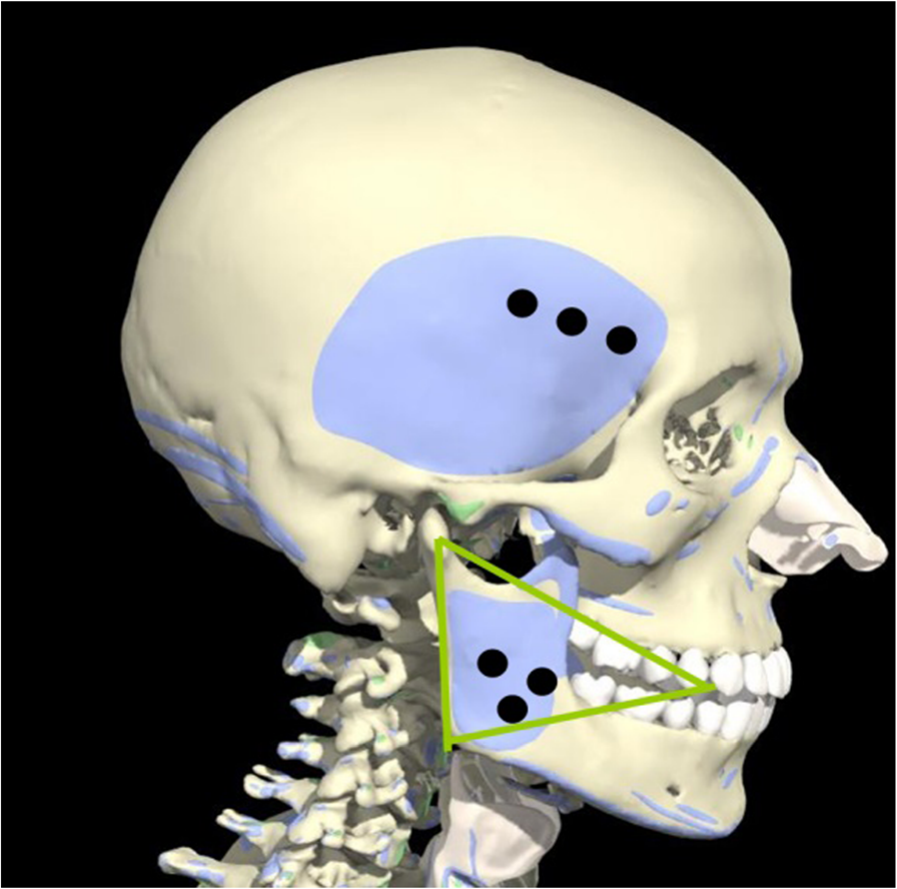
Areas colored with blue represent temporal and masseter muscles, black point represent injection point, and approximately 10 BU is injected to each injection point. Triangular area colored with yellowish green represent the landmark line of masseter muscle hypertrophy section

### Contraindication of botulinum toxin treatment

Major contraindication includes pregnancy, lactation, and musculoskeletal system disorder. Although clinical study for the effect of botulinum toxin on a fetus is not evident, Scott et al. had unofficially reported about nine pregnant women who had underwent Botox treatment during their pregnancy, among them eight women gave birth to a healthy infant, and only one woman gave birth to a premature infant. However, this premature delivery was not due to Botox treatment, and according to the latest report of Botox treatment in pregnant women, the women delivered a normal child [1]. Nevertheless, treating pregnant or lactating women with Botox is considered inappropriate. Several journals mention not to use botulinum toxin at any situations in children under the age of 12. Safety and efficacy of Botox in infant or children is not established, and muscle weakness is absolute contraindication because there is already damage in the function of neuromuscular junction and use of Botox might worsen the symptom [5, 10]. It is reasonable to say that there is almost no contraindication because it is difficult to diagnose myasthenia or musculoskeletal disorder for patients who visit a general dentist or a dental clinic

We have briefly discussed various indications, instructions of use, and contraindication as above. We will discuss the dental application of botulinum toxin in the next chapter, which includes theoretical and practical aspects of such as bruxism and square jaw.

## Treatment of bruxism using botulinum toxin

Several authors suggested that besides the flaccid paralysis and relaxation of muscles, which are the unique effects of botulinum toxin, other effects could be developed and used in more dental fields [2, 16–20]. If botulinum toxin can be used in combination with orthognathic surgery or esthetical surgery, it will be in the limelight as a drug maximizing the effect of the surgery.

The scope of treatment for a botulinum toxin can be broadly classified into three:

1. Treatment due to an effect of muscle relaxation of botulinum toxin that is flaccid paralysis of muscles caused by botulinum toxin, in other words, treatment due to the flaccid paralysis in the muscles responsible for wrinkles in the eyes and lines in the forehead.
2. Effect of muscle recession by botulinum toxin—effect of muscle recession due to flaccid paralysis of muscle by botulinum toxin, i.e., reducing the muscle mass by inducing the paralysis of muscles such as treatment of masticatory muscle or calf muscle.
3. Treatment using a pharmacological mechanism that reduces trigger points within the muscle fibers and reduces substances inducing pain such as substance P and serotonin released in muscle cells by effectively applying flaccid paralysis and muscle relaxation caused by botulinum toxin.

From the above-listed mechanisms, the third mechanism is the one presently getting the spotlight. This can be used very widely in the medical field, as well as in the dental field. It can be applied in various types of treatment including bruxism, tension headache due to temporomandibular joint disorder, and habitual subluxation of the temporomandibular joint [16, 19]. First and foremost, we would like to discuss the treatment of bruxism, of which the dentistry has a keen interest in.

### Definition and etiology of bruxism

Bruxism is a parafunctional habit that occurs during the day or in the night, sometimes clenching and grinding of teeth are all described as bruxism. Several causes are being reported for bruxism, and from an etiological point of view, psychological factor, emotional stress, and malocclusion are suspected as possible causes, but the exact cause remains unknown [21]. A perspective of regarding bruxism as a sleep disorder with a central nervous system origin rather than a disorder resulting from mere peripheral factors such as occlusal disharmony is dominant. Also, it is a well-known fact that post-traumatic bruxism can occur as sleep-wake cycle revives in patients who are in coma state due to brain damage [22]. Bruxism affects 5–96% of general population and its pathogenesis is not clearly known. It is not simply a central sleep disorder; there are several other contributing factors causing bruxism, therefore we are seeking a true solution through a series of symptomatic treatment.

### Symptoms and intra-oral conditions of patient with bruxism

Persistent contraction activity of masticatory muscles results in ischemia within the muscle cells, and this ischemia in turn promotes release of serotonin or pain-inducing substances from the surrounding tissues, and this pain mediator transmits pain from brain cortex to nerve endings. Feedback phenomenon transmitted to cerebral cortex leads to contraction of muscle and due to this vicious circle, it causes diseases such as spasm of muscles and myositis at the same time induces referred pain which results in the occurrence of series of symptoms such as migraine, stiffness in the cervical spine, and hypersensitivity of teeth. Also, it becomes the cause of persistent temporomandibular joint pain [4, 14, 15].

Botulinum toxin plays a role in breaking off this vicious circle resulting from persistent muscular contraction. It breaks off the feedback phenomenon by reducing the muscular contraction through flaccid paralysis, relaxing the muscles, and supplying blood to muscular tissue cells [18].

### Mechanism of treatment of bruxism using botulinum toxin

Botulinum toxin is a substance that has the ability to paralyze the muscles via inhibition of acetylcholine release at nerve terminal by acting on neuromuscular junction. Applying this, it reduces contraction of the muscle and lowers biting pressure up to 20–30% in the masticatory muscle. Botulinum toxin is expected to play many roles if its general pharmacological action is applied appropriately in dentistry. In particular, the use of botulinum toxin is emerging as a new alternative because many bruxism cases caused by occlusal disharmony have been treated with occlusion adjustments and the relief of bruxism was often observed when the cause of muscle contraction in the masticatory muscles was eliminated [1–4].

Since Van Zandijcke et al. (1990) first reported the therapeutic effect of Botox in the treatment of bruxism, it seems to be becoming a significant treatment method now [7]. Injecting into masseter muscle is generally more than enough for bruxism, and injecting into other masticatory muscles such as temporalis, medial and lateral pterygoid, digastric, geniohyoid is considered unnecessary. In fact, it has also been reported that this is the way to prevent deglutition disorder or severe masticatory functional disorder. Nevertheless, a common opinion of surgeons playing a pioneering role in the dentistry is that because temporal muscle plays a significant role in reducing bruxism phenomenon, it is better to inject botulinum toxin to both masseteric and temporal muscle if possible [19, 20].

### Conventional treatment method of bruxism

1. Control stress

Make the patient understand about the relationship between bruxism and stress and let him make the improvement by himself through a feedback system program and relaxation technique. Also, ask the patient to put an effort to reduce stress by improving his lifestyle.

2. Occlusal equilibration

Most of the patients with bruxism have prosthesis due to broken teeth or cracks, even though the teeth were free of caries, and often there is attrition on the occlusal surface of teeth and V-shaped grooves on the cervical area of teeth due to abfraction. In most cases, we are able to observe that patients with canine guidance occlusion changes to group functional guidance. In these patients, the transition from the group functional guidance to the canine guidance may be considered by eliminating the parafunctional occlusal pattern and removing the interference during lateral excursion or protrusion.

3. Splint therapy

We can expect the treatment of bruxism by improving the occlusal contact relationship, increasing the vertical occlusal dimension, and by extension of muscle spindles.

4. Drug therapy

Effect of muscle relaxants is minimal, and there is a report on the usage of antidepressants, since bruxism is recognized as sleep disorder disease. Combination therapy seems to be effective.

### Treatment of bruxism using botulinum toxin

We had a case with a patient whose bruxism phenomenon had greatly reduced when we injected botulinum toxin to each or both masseteric and temporalis muscles. In the case of a patient who did not respond to occlusal equilibration and splint therapy, botulinum toxin remarkably reduced the bruxism phenomenon. In the literature, especially Ivanhoe et al. reported that botulinum toxin could temporarily stop the serious symptoms of bruxism for a period of 3 months in a patient with brain damage due to cardiac arrest. With such case report, it is evident that botulinum toxin exhibits uniform therapeutic effect on bruxism patients [18].

According to To et al., masseteric muscle mass was reduced by 31% on ultrasonic and electromyogram 3 months after the injection. They reported that out of nine masseter muscles used in the test, six maintained atrophic state for 1 year [14]. Therefore, we can assumed that reduced biting force due to atrophy of masseter muscles aid in prevention of bruxism by temporarily inducing environmental change in the occlusion. Generally, biting force is known to decrease up to 20–40%; because muscle relaxation more effectively progresses at resting, it is known to reduce excessive clenching of teeth or bruxism phenomenon during the sleep.

**Table:** 

**Fig. 11:**
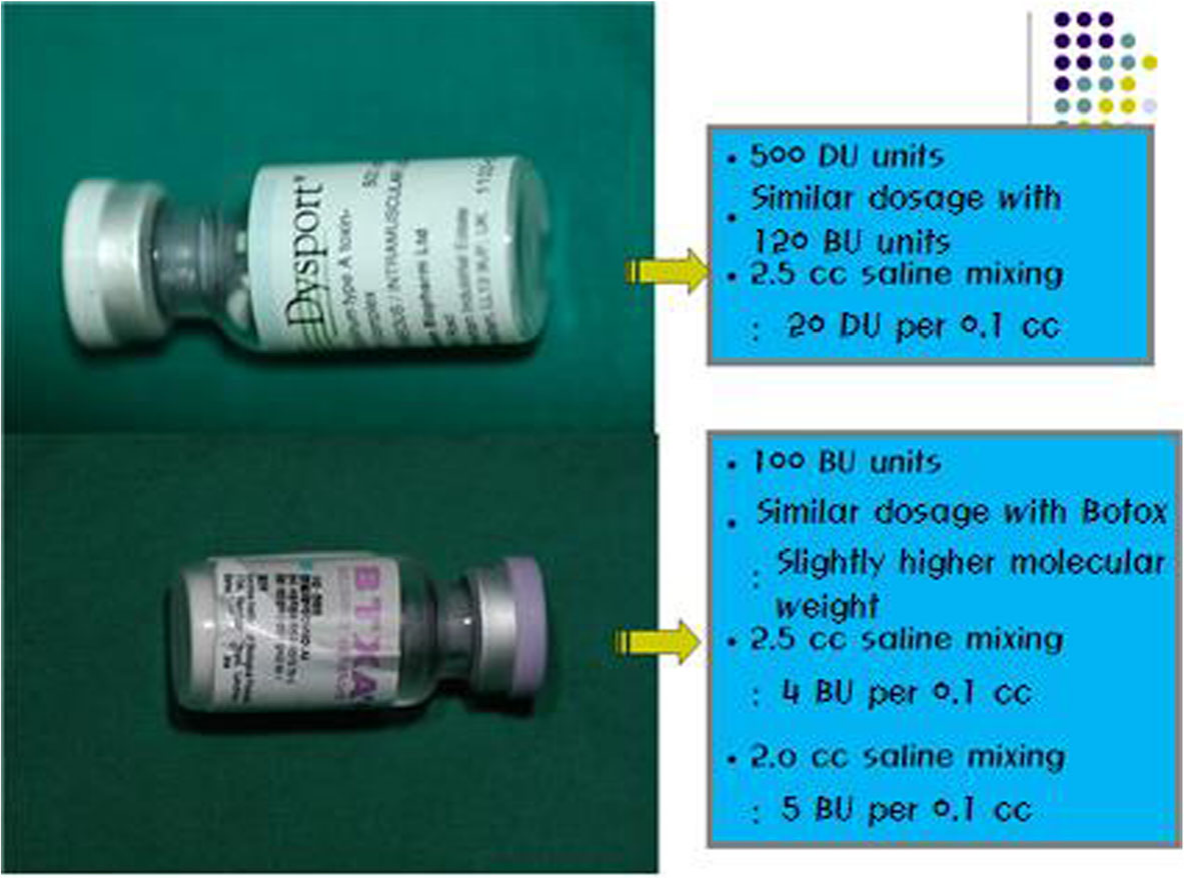
Brand names for botulinum toxin A include Dysport, BTXA, Botox, etc. The drugs differ from each other in their unit and possess different methods of injection depending on their method of dilution

[Based on the clinical experience of the present writer] (Figs. 10 and 11) A dose for masseter muscle and temporal muscle in the treatment of bruxism. 1) Masseter muscle: 25–30 BU (BTXA, unit of Botox) per one side 2) Temporal muscle: 15–20 BU (BTXA) per one side	

### Treatment of bruxism—case

**Fig. 12:**
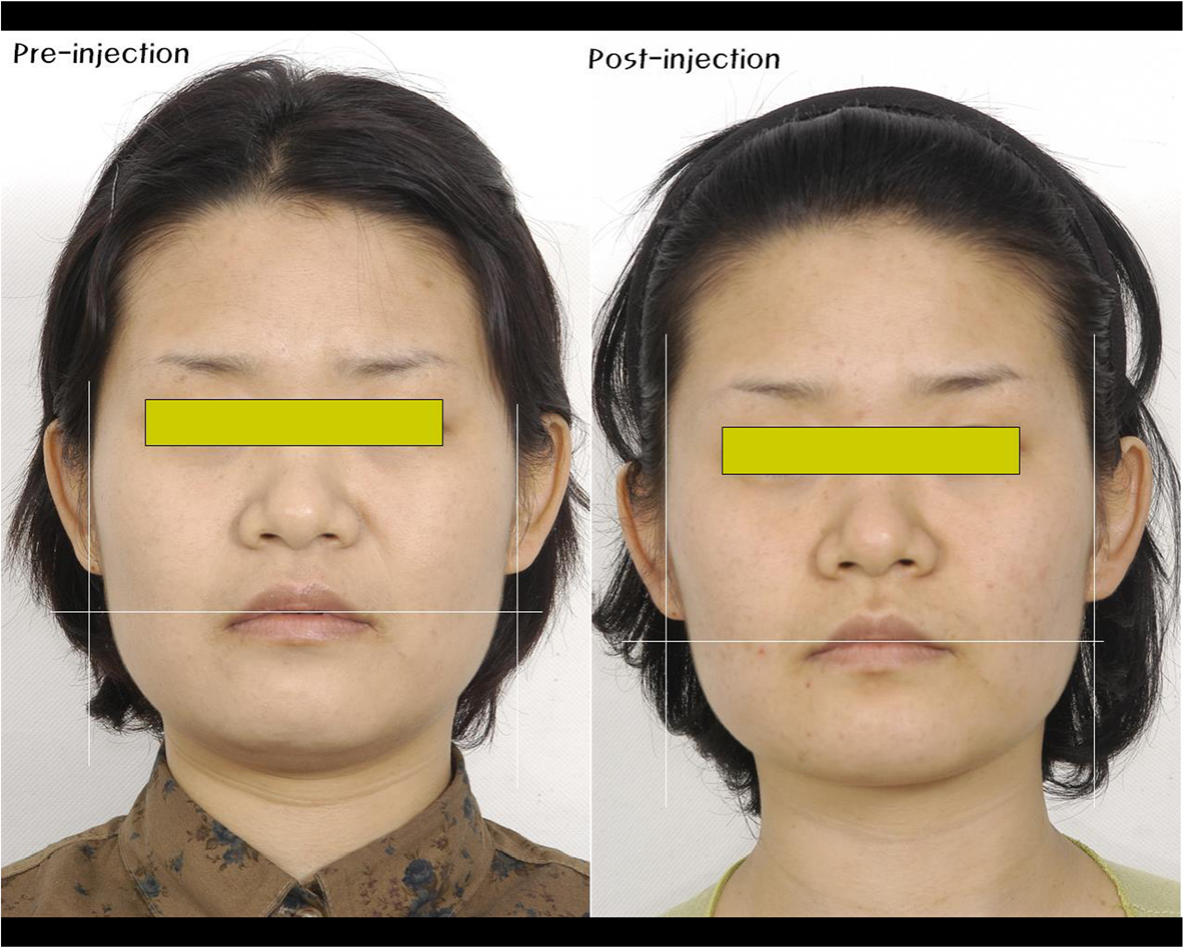
A patient who had chronic migraine, bruxism, benign masseteric hypertrophy. Pre-injection of BTX (left), after 1 month clinical photo (right)

In our case, we initially perform both occlusal correction and splint therapy, and then we prescribe the patients with antidepressants and while watching the progress of the disease, we inject them with botulinum toxin (BTXA, Dysport). In Fig. 12, the patient had bruxism, chronic migraine, and bilateral benign masseteric hypertrophy (BMH). When we injected 30U BTXA (Hanall pharmaceuticals) into the left and right side of masseter muscle to treat bruxism and BMH, BMH symptom considerably improved and bruxism was also resolved after a month. We were able to observe the significant reduction of referred pain as the area of trigger point caused by ischemia due to muscle contraction disappears. After 6 months of initial injection, the patient received the second injection with the same dose as the initial one, symptoms of bruxism resolved continuously.

**Fig. 13:**
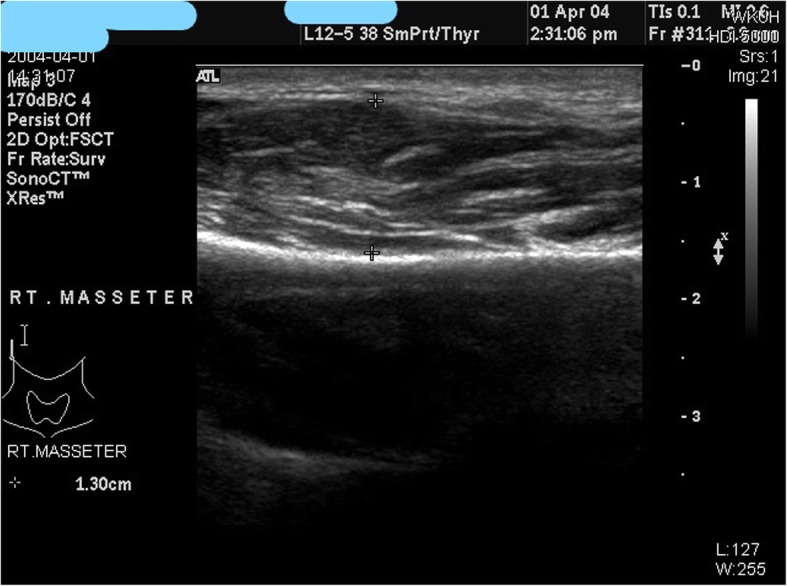
Diagnosing method for square jaw: sonograph. With the help of Panorama, skull PA, and sonograph of masseter muscle, you can identify the skeletal portion and patterns of muscle hypertrophy, and able to decide which one to use: surgical intervention or botulinum toxin injection

**Fig. 14:**
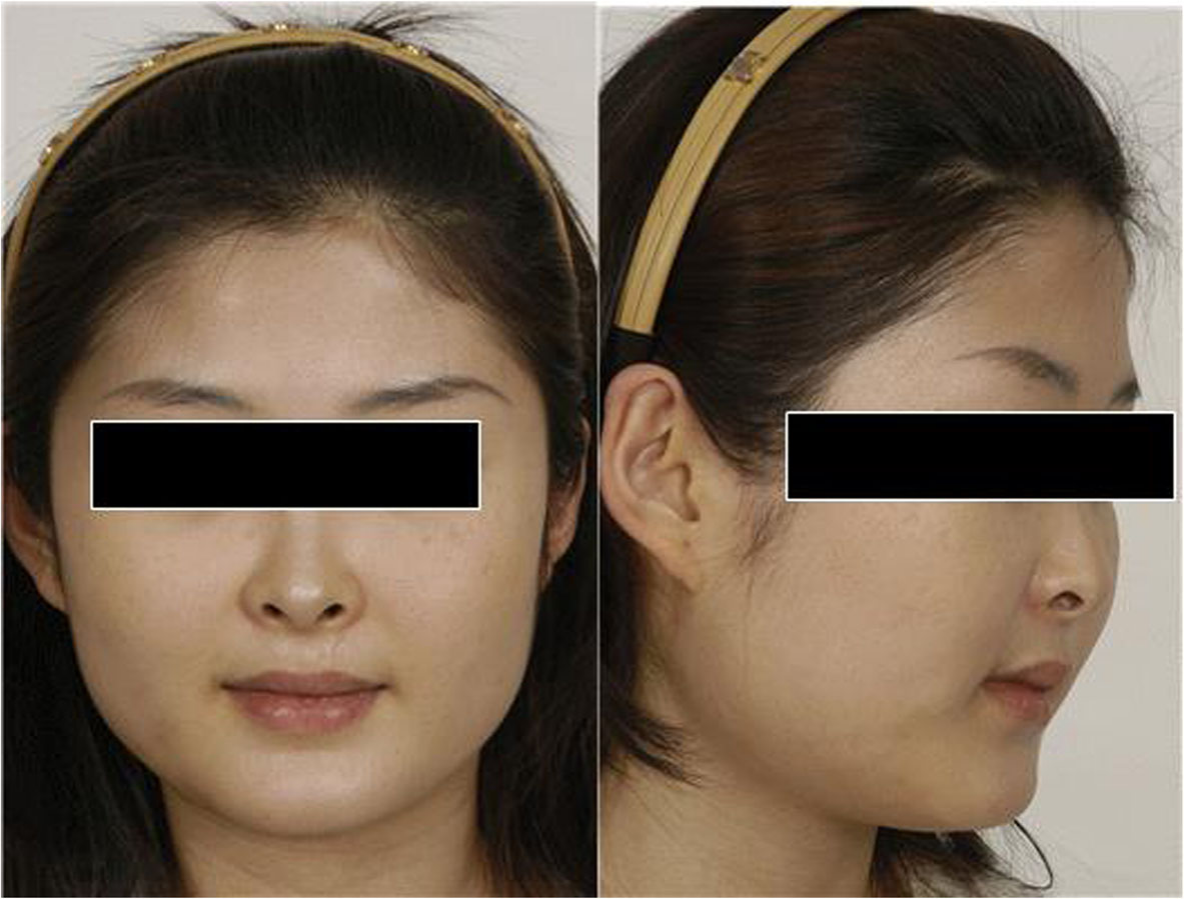
Type I. muscular type. Hypertrophy of masseteric muscles is prominent, improvement is expected after botulinum toxin A injection

**Fig. 15:**
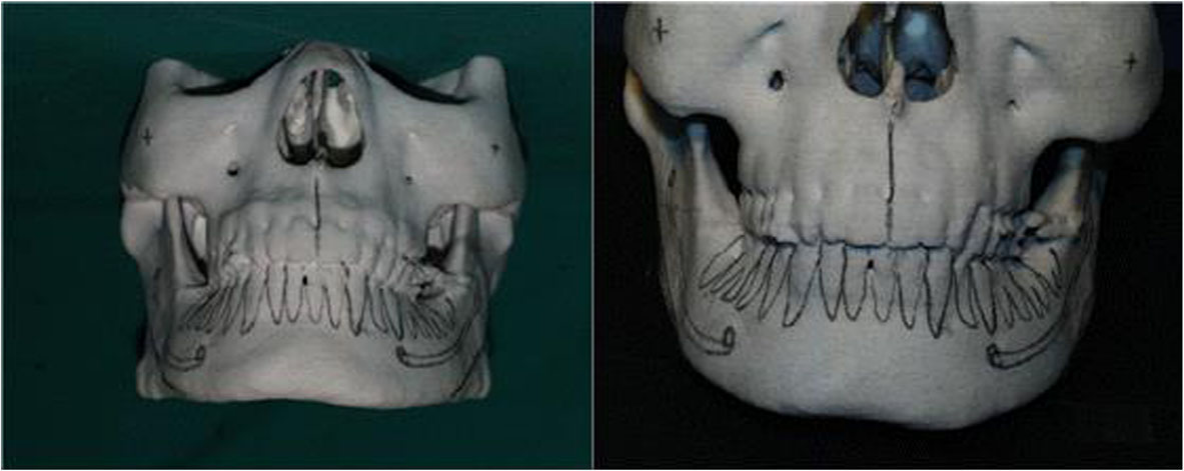
Type II. Skeletal lateral projection type. Picture in the left is pre-operative 3D model, picture in the right is the post-operative picture directly performed on 3D. Angle reduction is noticed

**Fig. 16:**
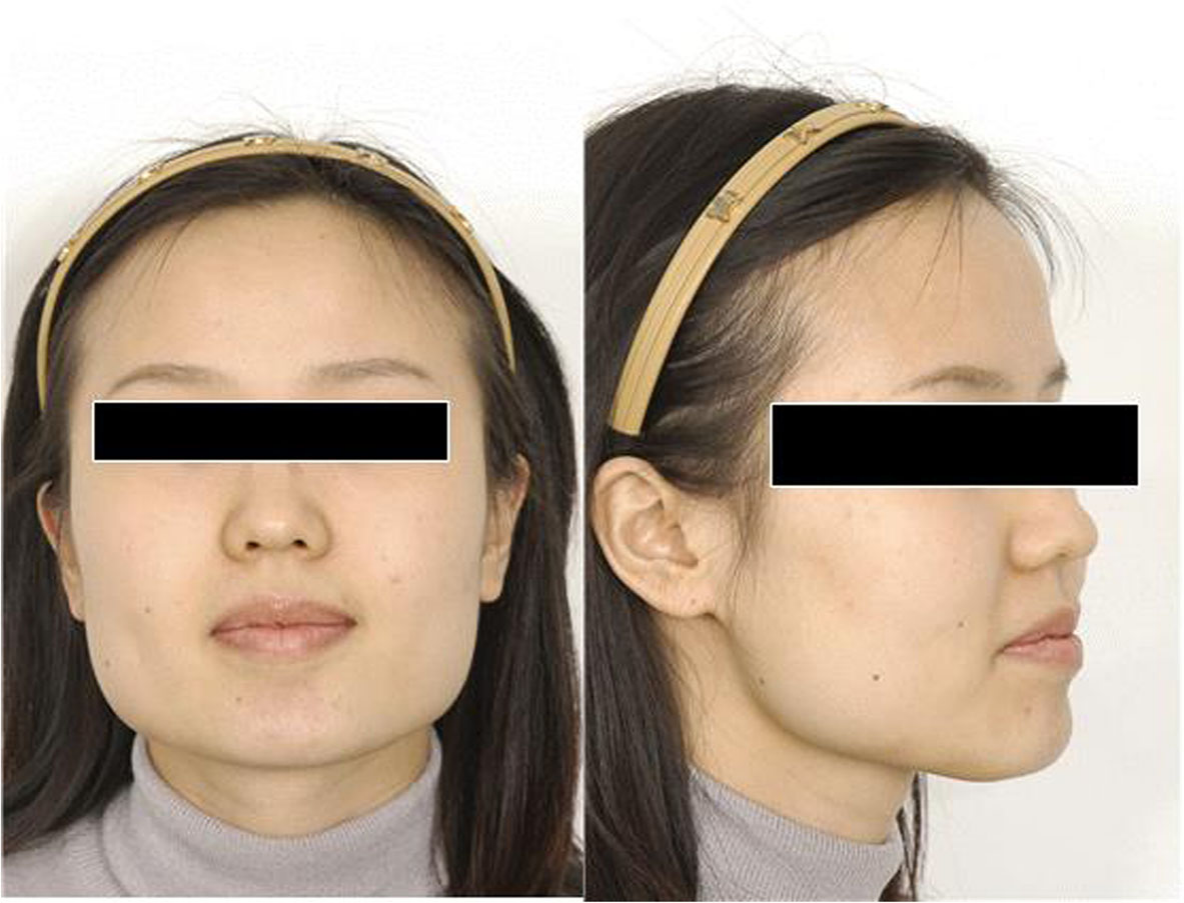
Type III. Combination type. Patient with outwardly projecting mandibular angle and muscular hypertrophy is observed

Since the side effect is minimum, there is not much to worry about. Transient difficulty in mastication is possible but it naturally resolves after a week. Therapeutic effect on masseter muscle starts to appear after 3 weeks, therefore surgeon needs to clarify about this at the time of consultation with the patient or his guardian. The patient, in this case, complained that had it been over a week and still there was no treatment effect. After 3 weeks, as the symptoms of square jaw and clenching started to resolve, he was very satisfied with the treatment at last.

### Precaution for botulinum toxin injection in bruxism patient

The masseter muscle is located near the facial nerves and is likely to weaken the seventh nerve that is responsible for facial muscles when the toxin is injected into this area. Therefore, to use toxin effectively with minimum side effects, it is important to inject the toxin into the center of the triangle as we mentioned earlier. Generally, injecting botulinum toxin into the muscle can reduce muscle mass by about 30% and has a sufficient effect for treatment of muscle hypertrophy.

Although several authors have different opinions about the temporal muscle, by referring to the recent trends, we can assume that injecting into the masseter muscle along with temporal muscle will reduce muscle activity and result in flaccid paralysis of the muscles. As much as masseter muscle is considered important, the temporal muscle plays significant role, so it is a good idea to inject into both muscles simultaneously. It is crucial not to inject into the vessel by mistake via aspiration and to avoid injecting into lateral canthus because there is a possibility of paralysis during eye movement and eyelid exercise [20].

## Treatment of patient with square jaw using botulinum toxin

Use of botulinum toxin is gaining popularity and its procedure is developing in various ways as well, and at present, square jaw reduction is the procedure that oral maxillofacial surgery has a keen interest in. Particularly, our country has more interest than any other countries in the world. Especially when we observe the appearance of face of Asians, well-developed lower jaw and its shorter vertical length contribute to prominent appearance of a square jaw. Moreover, the square jaw appears to be more prominent in Asians because they have a lower nose bridge and smaller eyes when compared with Westerners. In Korea, since a woman with a slender jawline is considered a beauty, the angular jawline may become one of the inferiority complexes especially for women in their 20s and 40s.

It is quite true that the number of doctors performing square jaw reduction keeps increasing because the procedure is not as difficult as the common anti-wrinkle treatment in its principle or procedure. However, it should be remembered that square jaw reduction using botulinum toxin is just an auxiliary method to surgery. In other words, square jaw cannot be treated solely with botulinum toxin.

In our case, we treated square jaw patient by dividing the treatment plan into three methods and had set up a treatment plan by dividing the form of the square jaw into three types. We normally divide the treatment into three methods: treating with only botulinum toxin, combination therapy with botulinum toxin and square jaw reduction, and treatment with square jaw reduction alone. We can make the treatment plan based on sonograph, skull PA, and panorama. Combination therapy with botulinum toxin and square jaw reduction (mandibular angle contouring surgery) accounts for 60% and botulinum treatment alone accounts for 30% of the total treatment.

### Definition of square jaw (benign masseteric hypertrophy) and background of treatment

In English, benign masseteric hypertrophy is also referred to as a squared face or square jaw. It is called so because lower jaw is thickened bilaterally and anteroposteriorly from the frontal and lateral view respectively, and that makes the face appear square in shape. In terms of maxillofacial deformity, square jaw is divided into two forms: one is benign masseteric hypertrophy or simply masseteric hypertrophy, a state where the muscles are enlarged, and the other is prominent mandibular bone, square shape mandible, square mandible face, and prominent mandibular angle, where the bony part is enlarged. Mixed type of both forms is also often observed in clinical settings. While Western mentions that hypertrophy of masseteric muscle is the cause of square jaw, Asia claims that large bone is the main reason for a square jaw. However, in our opinion, both can be the reasons for the square jaw, and unless both causes are resolved, it is difficult to bring about change in both frontal and lateral view.

The square jaw can be corrected by surgical intervention for the case present with outwardly protruding mandible and hypertrophy of masticatory muscles, which are of both skeletal and muscular problems. In this article, correction of the square jaw using botulinum toxin is recommended for patients with prominent benign masseteric muscle hypertrophy, and it is recommended for the patient whose hypertrophy phenomenon remains due to muscular problems even after successful square jaw reduction surgery.

In 1880, Dr. Legg was the first to report benign masseteric muscle hypertrophy in a 10-year-old girl who had no special family history or abnormal eating habits, and since then several similar cases were reported intermittently. Gunery performed an operation with an intraoral approach for the first time in 1947, and usefulness of the operation was recognized by several other researchers. Then, in 1994, since Smyth and Moore et al. simultaneously announced that treatment of benign masseteric hypertrophy using Botox to be very effective, it is introduced as a method that is enthusiastically recommended in Korea [8].

In 1990, Schnider et al. had reported that when they injected botulinum toxin into the masseter muscle, which is target muscle, significant muscle atrophy occurred for 3 to 8 weeks that remained for 25 months of the follow-up period and no side effect was observed. Since 2001, it is supplied to various fields in Korea such as oral maxillofacial surgery, dermatology, plastic surgery, and orthopedics, and its effects are being evaluated in various fields [11, 20].

### Clinical anatomy of masseter muscle and periangular area

Anatomically, masseter muscle is a rectangular-shaped thick and powerful masticatory muscle, where its superficial portion originates from the zygomatic process of maxilla and anterior 2/3 of a lower portion of the zygomatic arch, descends posteriorly and ends at a mandibular angle and lower 1/2 of the lateral surface of mandibular ramus. On the other hand, deep portion originates from medical surface of zygomatic arch, ends at upper 1/2 of ramus and a lateral surface of the coronoid process.

Masseteric muscle is innervated by the mandibular branch of the trigeminal nerve, and blood is supplied by the masseteric branch of the maxillary artery. The masseteric fascia or parotideomassetric fascia is strongly attached to the masseteric muscle at the same time as it covers the masseteric muscle. It is attached to the bottom of the zygomatic arch superiorly, and posteriorly it is attached to the parotid gland.

### Treatment method based on types of square jaw

We proceed with the treatment by classifying the protrusion of mandibular angle into three types based on the cause. As mentioned above, we determine the pattern of treatment based on causes of protrusion of mandibular angle, i.e., muscular problem or bony problem or both. This judgment is made based on Panorama, skull PA, and muscle sonography (Fig. 13).

Type I is protrusion of mandibular angle mainly due to hypertrophy of masseteric muscle, which is considered normal in terms of its bone structure, and the mandibular angle appears protruding when viewed from the front, and appears normal when viewed from lateral (Fig. 14). In this case, Botulinum toxin A (BTXA ®, Hanall pharmaceuticals) is used rather than a surgical method, and it becomes an indication that can provide a good therapeutic effect. In this case, patient benefits from Botulinum toxin A injection alone and therefore does not need to consider additional surgical intervention. More than 10 mm of hypertrophy of masseter muscle is observed in sonograph, and outward projection in mandibular angle is not observed on Panorama and skull PA. This phenomenon is also observed in the case with the posterior projection of mandibular angle. The patient benefits from botulinum toxin injection in such cases. However, since the change in the lateral aspect is difficult to achieve, additional surgical intervention are considered, which patient may not think it is necessary.

Type II is the case where thickening of mandibular angle occurs mainly due to the protrusion of the mandibular angle, and hypertrophy of masseteric muscles is generally not observed much (Fig. 15). Botulinum toxin A injection is used as an adjunct to square jaw reduction as it can contour the remaining fullness of the face. When square jaw reduction is performed and postoperative edema remains, patients may complain that they cannott find a difference before and after the surgery. In such cases, they can benefit from adjunctive botulinum toxin A injection.

Type III is mainly due to both thick mandibular angle and hypertrophy of masseteric muscles, and the protrusion of mandibular angle can be observed both in the frontal and lateral view (Fig. 16). Both muscle and bone being the cause, it can be resolved by surgery and botulinum toxin injection. Strong impression in the appearance of face remains if only muscular problems are resolved.

According to Baek et al., when they divided patient with a square face into three types, i.e., lateral bulging in frontal view, posteroinferior projection in lateral view, and mixed type, they accounted for 41%, 16%, and 44% respectively. In clinical settings, in the case of 44%(mixed type), along with surgical intervention, use of botulinum toxin A injection is expected to provide a good effect.

## Conclusion

Bruxism and square jaw are the most common indications for botulinum toxin injection. For square jaw, inject 8–10 BU per point into masseteric muscle at 3 to 4 points. A dose for masseter muscle is 25–30 BU and temporal muscle is 15–20 BU per one side in the treatment of bruxism. Botulinum toxin corrects the patterns of muscle exercises, decreases square jaw, and alleviates pain by changing the patient's lifestyle. More verification should be made on muscle contraction and muscle activity, and various approaches should be made for the maxillofacial field.

## Data Availability

Not applicable (data sharing not applicable to this article as no datasets were generated or analyzed during the current study).

## Abbreviations

BU: Botox unit
DU: Dysport unit
BMH: Benign masseteric hypertrophy
